# Mendelian randomization analysis: The causal relationship between C-reactive protein and amyloidosis and between C-reactive protein and atherosclerosis

**DOI:** 10.1371/journal.pone.0329612

**Published:** 2025-08-08

**Authors:** Chunhui Liu, Xupeng Huang, Yongsheng Huang, Hongguang Jin

**Affiliations:** 1 Changchun University of Traditional Chinese Medicine, Changchun, China; 2 Hospital Affiliated to Changchun University of Traditional Chinese Medicine, Changchun, China; Johns Hopkins: Johns Hopkins University, UNITED STATES OF AMERICA

## Abstract

**Background:**

A number of studies have shown that elevated CRP is linked to AS and reduced CRP is linked to amyloidosis. However, the exact mechanism explaining this connection is not known.

**Methods:**

We used genomic pooled data from the Genome-Wide Association Study (GWAS) in a two-sample, two-way Mendelian randomization (MR) analysis study. Methods used included inverse variance weighting (IVW), weighted median (WM), MR-Egger method, Cochran’s Q, MR-PRESSO, MR-Egger intercept test, and leave-one-out sensitivity analysis. To investigate the specific causal relationship between C-reactive protein and amyloidosis and between C-reactive protein and atherosclerosis (coronary, cerebral, aortic, and peripheral atherosclerosis). The study procedure was performed with the STROBE-MR checklist.

**Results:**

There was a inverse association between C-reactive protein and amyloidosis and an positive causal relationship between C-reactive protein and aortic atherosclerosis. The development of aortic atherosclerosis was positively correlated with C-reactive protein levels (IVW:p = 0.003, OR=1.203,95% CI:1.066–1.358). Whereas amyloidosis onset was associated with reduced C-reactive protein levels (IVW:p = 0.022, OR=0.582,95% CI:0.366–0.924). Reverse Mendelian randomization analysis found no evidence of reverse causality.

**Conclusion:**

We verified the existence of a negative association between C-reactive protein and amyloidosis and a positive association between C-reactive protein and atherosclerosis by Mendelian randomization, which may provide some reference value for subsequent studies and treatment in the clinic.

## 1. Introduction

Amyloidosis is an infiltrative disease with lesions that can spread diffusely throughout the body [1]. It encompasses multiple types classified by etiology. Primary amyloidosis (AL type) is a plasma cell disorder where abnormal monoclonal plasma cells generate excessive amounts of immunoglobulin light chains [2]. These light chains misfold and form amyloid proteins that are deposited, commonly affecting organs such as the kidneys, heart, and nervous system. Secondary amyloidosis (AA type) frequently occurs secondary to chronic inflammatory diseases, chronic infections, or malignant tumors [3,4]. In this case, an increase in serum amyloid A (SAA) leads to the production and deposition of AA proteins, mainly involving organs like the kidneys, liver, and spleen [5]. Hereditary amyloidosis results from gene mutations that alter the structure of proteins, such as transthyretin (TTR), subsequently leading to the deposition of amyloid fibrils, often involving the nervous system and heart [6]. Dialysis-related amyloidosis (β₂ - microglobulin amyloidosis) is specific to long-term dialysis patients. Due to incomplete clearance of β₂ - microglobulin by dialysis membranes, it accumulates and deposits predominantly in joints and bones, causing corresponding symptoms [7]. The pathological changes are mainly characterized by amyloidosis of muscle fibers [8]. Insoluble and misfolded proteins accumulate not only in local tissues but also in peripheral tissues, triggering either local or systemic lesions [9]. According to a survey in the United States, the prevalence of the disease has increased significantly from 2007 to 2015, having risen from 15.5 cases per million to 40.5 cases per million [10]. A 2014 study on patients diagnosed with stage III cardiac amyloidosis at the Mayo Clinic revealed that the 1-year survival rate of such patients was only 25% [11]. Moreover, they are extremely sensitive to the toxicity of therapeutic drugs, which can cause further damage to the heart and thus lead to a very poor prognosis, and for patients with compromised cardiac function, without aggressive treatment, their survival period can be shortened to approximately 6 months [12]. The disease can affect peripheral tissues and organs throughout the body, and the damage to the heart and kidneys, in particular, is often the leading cause of death among patients [13]. Cardiac amyloidosis is one of the severe manifestations. Although such cases are relatively rare, the harm it poses to the patients lives cannot be overlooked [14]. Previous research has indicated that the disease is associated with genetic factors, and gene locus mutations are a common cause. It is caused by the accumulation of misfolded transthyretin proteins within the myocardium, which subsequently leads to amyloid changes in the myocardium [15]. Currently, over 150 different pathological point mutations have been identified in the transthyretin gene [16]. Moreover, the mutation sites vary among different ethnic groups. The common cause among African and Caribbean populations is the mutation at the Val122Ile site, while the V30M mutation is prevalent in the Caucasian population [17]. However, it is not clearly stated in the publicly available data how many single nucleotide polymorphism sites (SNPs) among these mutation sites are significant in genome-wide association study (GWAS) loci [18]. A study by Gwyther, M., et al. in 1982 discovered that C-reactive protein (CRP) levels were significantly elevated in young children with chronic osteoarthritis who developed amyloidosis between the ages of 5 and 15 years, and this elevated level could persist for months to years [19]. A study by Falck et al. also confirmed that CRP is closely related to amyloidosis, and the clinical measurement of CRP levels has a certain reference value in assessing amyloidosis. Through linear regression analysis, they found that there was a significant correlation between the concentrations of serum amyloid A and C – reactive protein (r = 0.81, p < 0.001) [20]. However, the specific causal relationship between the two is still unclear.

Atherosclerosis (AS), classified by the types of affected blood vessels, includes coronary, cerebral, and peripheral atherosclerosis [21]. Coronary atherosclerosis often leads to coronary heart disease, severely threatening heart health. Cerebral atherosclerosis is prone to causing stroke, dealing a devastating blow to nervous system functions. Peripheral atherosclerosis affects limb blood supply, resulting in symptoms such as intermittent claudication. AS is a disease mainly mediated by an inflammatory response. Its pathological changes are due to the action of inflammatory cells and inflammatory molecules in the arterial wall, which produce plaques [22] that impede or even block the blood flow within arteries. The disease can affect the peripheral arterial vessels, with cardiovascular and cerebrovascular vessels being the most commonly involved clinically [23]. The cardiovascular and cerebrovascular diseases induced by AS are the main causes of death, imposing an economic and health burden on the global population [24]. CRP is a plasma protein that is closely related to the process of inflammation and it is frequently used as an indicator for monitoring inflammatory response in clinical practice [25,26]. Research has found that CRP can interact with cholesterol, acquire lipid-like flocculent properties, and bind to modified low-density lipoprotein (LDL) in vivo, thus promoting the formation process of AS [27–29]. However, the specific effects and causal relationships between them remain unclear and require further research for confirmation.

Low-density lipoprotein cholesterol (LDL-C) plays a crucial role in the pathogenesis of atherosclerosis (AS) and is one of the significant risk factors for the development of AS [30]. Meanwhile, C-reactive protein (CRP), as a marker of inflammatory response, is also closely related to the degree of inflammation in the process of AS [31]. In recent years, exploring whether LDL-C can serve as a mediator variable between CRP and AS for Mendelian randomization analysis has become a research hotspot. From a biological mechanism perspective, LDL-C may indirectly affect CRP levels through various pathways and thus mediate between CRP and AS. On the one hand, high LDL-C levels can trigger a series of pathological processes accompanied by an inflammatory response, leading to an increase in CRP levels. On the other hand, LDL-C may directly or indirectly influence the function of inflammatory cells and the production of inflammatory factors, thereby regulating CRP expression. For example, oxidized LDL (ox-LDL) can activate intracellular signaling pathways in inflammatory cells, promote the release of inflammatory factors, and subsequently stimulate the liver to synthesize CRP [32]. However, determining whether LDL-C is a mediator variable between CRP and AS for Mendelian randomization analysis still faces many challenges. Firstly, it is necessary to identify appropriate genetic instrumental variables related to LDL-C that meet the assumptions of Mendelian randomization, i.e., having a strong association with the exposure factor (LDL-C) and only affecting the outcome (AS) through the exposure factor without pleiotropic effects and other issues. However, among the currently known genetic variants related to LDL-C, some may have pleiotropic effects, not only affecting LDL-C levels but also potentially directly affecting CRP or AS, which will interfere with accurately determining the mediating effect [33]. In addition, the linkage disequilibrium phenomenon among genetic variants may also affect the validity of the instrumental variables [34]. Simultaneously, conducting such an analysis requires a sufficiently large sample size and high-quality data to accurately detect potential causal relationships. Especially when exploring complex relationships such as the mediating effect, higher requirements are placed on the sample size and data quality. If the sample size is insufficient or the data quality is poor, it may lead to unstable results and false negative or false positive conclusions, thereby affecting the accurate assessment of the mediating role of LDL-C. Therefore, although theoretically exploring LDL-C as a mediator variable between CRP and AS for Mendelian randomization analysis is of great significance, in practical operations, it is necessary to carefully consider and address the aforementioned numerous problems to ensure the reliability and scientific nature of the research results.

The reasons for selecting amyloidosis and atherosclerosis (AS) for the Mendelian randomization study are as follows. Firstly, both diseases are highly harmful clinically. Amyloidosis affects tissues and organs throughout the body, especially causing severe damage to the heart and kidneys, endangering lives. AS-induced cardiovascular and cerebrovascular diseases are important factors contributing to global mortality, imposing a heavy health and economic burden on society [35]. Secondly, previous research has shown that CRP is closely related to both diseases. In amyloidosis, patients’ CRP levels change significantly, and CRP has a reference value for assessing the disease condition. In AS, CRP has been found to interact with cholesterol and other substances to promote the disease process [36]. However, the specific causal relationships remain unclear. Through Mendelian randomization studies, using genetic variation as a natural randomization tool, we can effectively overcome the interference of confounding factors and reverse causality in traditional observational studies, accurately exploring the exact causal association between CRP and amyloidosis, and between CRP and AS, providing a solid basis for the subsequent formulation of targeted diagnosis and treatment strategies. Therefore, this Mendelian randomization study aims to explore the specific causal association between CRP and amyloidosis and between CRP and AS.

## 2. Materials and methods

### 2.1. Study design

**Fig 1:**
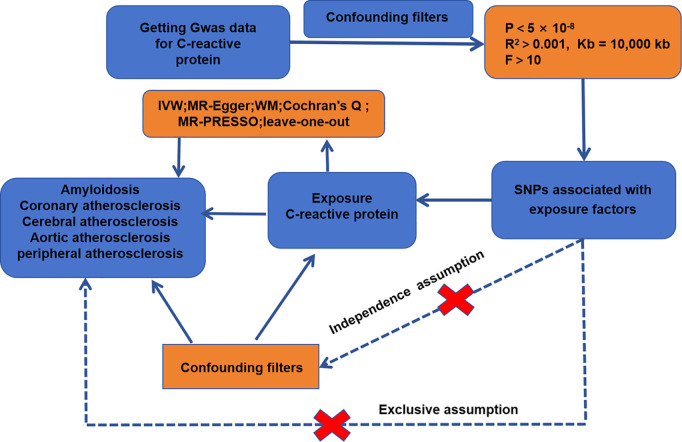
Mendelian randomization assumptions and study flowchart. Illustrates the three core assumptions of Mendelian randomization (correlation, exclusivity, independence) and the study design flow. Key elements: Instrumental variable selection criteria; exposure (CRP) and outcome (amyloidosis, atherosclerosis subtypes) relationships; analysis workflow.

The main objective of this study was to investigate the causal association between CRP and amyloidosis and between CRP and AS (including coronary, cerebral, aortic, and peripheral atherosclerosis), and we carried out a bidirectional, two-sample, Mendelian randomization study. We performed an initial screening of included instrumental variables (IVs) for single nucleotide polymorphisms (SNPs) with a strong correlation with exposure factors. The study methodology complied with the requirements in the STROBE-MR checklist, and three basic assumptions have been fulfilled, i.e., the assumption of correlation, the assumption of exclusivity, and the assumption of independence [30,31]. The specific process is shown in Fig 1.

In the correlation assumption, SNPs with a strong correlation with exposure were screened based on a threshold of less than 5 × 10^−8^ for P. The exclusion assumption, i.e., counteracting linkage disequilibrium and controlling for a threshold of pairwise correlation (r2) greater than 0.001 and the number of base pairs (Kb) = 10,000 kb. The independence assumption excluded other confounding factors that interfered with the relationship between the exposure and the outcome.

### 2.2. Data acquisition

The single nucleotide polymorphisms (SNPs) associated with CRP in this study were obtained from the Genome-Wide Association Study (GWAS) meta-analysis dataset provided by the United Kingdom Biobank (UKBB), which can be accessed at https://gwas.mrcieu.ac.uk. A total sample size of 353,466 samples was included in the analysis, which involved 19,057,467 SNPs. Genetic data related to amyloidosis and AS were obtained from the GWAS database, with amyloidosis data from 197,485 individuals (covering 226 cases and 197,259 controls) and 16,380,377 SNPs, and AS data: coronary atherosclerosis data with 361,194 samples (14,334 cases, 346,860 controls) and 13,586,589 SNPs, cerebral atherosclerosis data with 218,792 samples (104 cases, 218,688 controls) and 16,380,466 SNPs, and aortic atherosclerosis data with 150,765 samples (4,373 cases, 406,111 controls) and 7,992,739 SNPs. The peripheral atherosclerosis data consisted of 168,832 samples (6,631 cases, 162,201 controls) and 16,380,247 SNPs. The sample sizes in the present study were from European populations. It is worth noting that the subjects in the original studies for which the data were collected gave written consent and that all the study protocols were theoretically approved by their respective review boards. Therefore, no additional ethical approvals or licenses were required for this MR study.

### 2.3. Screening of instrumental variables

The final desired SNPs were obtained by making P < 5 × 10^−8^, thus screening the IVs that showed strong association with CRPs after the following process: (1) relevant SNPs were extracted online from the GWAS summary data using the TwoSample MR package in the R 4.3.2 software, and (2) the genomic distances of the SNP manifestation r^2^ within 1,000 base pairs (Kb) were greater than 0.001 to address linkage disequilibrium; (3) SNPs with overlapping or alleles that could not be corresponded to were excluded to prevent impact on the analysis results; (4) http://www.phenoscanner.medschl.cam.ac.uk/ was utilized to eliminate the effect of confounding factors [32]; (5) using the statistic F to detect the presence of bias in instrumental variables [33], taking a threshold of F > 10 for identification, with the relevant formula F = [(n-k-1)/k] * [R^2^/(1-R^2^)], where n denotes the sample size and k denotes the IV number. R^2^ denotes the proportion of genetically related exposure variants. If the F value was less than 10, it indicated that the SNP in question was a weak instrumental variable and was excluded from this analysis to attenuate the bias caused by the results of this study.

### 2.4. Statistical analysis

The two-sample two-way Mendelian randomization analysis was used in this study. The research process was carried out using the TwoSampleMR and MR-PRESSO packages in the R 4.3.2 software [34]. The main methods used included the inverse variance weighted (IVW) method, the weighted median (WM) method, and the MR-Egger of which the IVW method is considered to be the causality determination main basis, in which the weighted mean is weighted by the inverse of the variance of each IV to ensure the validity of the IV. The corresponding P-value <0.05 is considered significant for causality. To make the results more robust, in addition to the IVW method, we also used MR-Egger and WM analyses as auxiliary tools. MR-Egger intercept test and outliers (MR-PRESSO) were used to test for the presence of horizontal pleiotropy during the test period [37,38]. A significant MR-Egger intercept is significant for horizontal pleiotropy, and a P-value of 0.05 was used as the threshold, and a P-value less than this value is considered significant if there is horizontal pleiotropy [37,38] and MR-PRESSO global analysis test was applied to SNPs to eliminate horizontal pleiotropy. Heterogeneity was assessed by Cochran’s Q test [38], where P < 0.05 indicated the presence of heterogeneity, and a random effects model was applied, and vice versa for a fixed effects model. Causality was assessed using the ratio of ratios (OR) and 95% confidence intervals (CI). If the OR was < 1, the exposure was protective of the outcome, and vice versa, it posed a risk to the outcome. At the end of the analysis, we performed a reverse MR analysis with the same parameter settings and data samples. Finally, we used the RStudio software platform to visualize the results of the analysis in the form of forest plots, scatter plots, and leave-one-out plots.

## 3. Results

### 3.1. IV screening results

The sample datasets in this study were all from the GWAS database, and the required SNPs were screened as IVs with a P-value less than 5 × 10^−8^, and the F-statistic of each IV was greater than 10, indicating that there was no weak bias in the IV results. The relevant SNP results are shown in Table 1 in the Supplementary Material.

**Table 1:** Results related to MR analysis.

Exposure	Outcome	Inverse variance weighted	P_value	MR-Egger	P_value	Weight Median	P_value
		OR(95%CI)		OR(95%CI)		OR(95%CI)	
**CRP**	**Amyloidosis**	**0.582(0.366-0.924)**	**0.022**	**0.559(0.300-1.042)**	**0.092**	**0.385(0.184-0.807)**	**0.011**
**CRP**	**Coronary AS**	**0.998(0.995-1.002)**	**0.400**	**0.992(0.987-0.997)**	**0.003**	**1.000(0.994-1.004)**	**0.591**
**CRP**	**Cerebral AS**	**1.029(0.484-2.187)**	**0.941**	**1.210(0.436-3.358)**	**0.715**	**1.168(0.358-3.815)**	**0.797**
**CRP**	**Aortic AS**	**1.203(1.066-1.358)**	**0.003**	**1.107(0.937-1.306)**	**0.234**	**1.198(1.001-1.434)**	**0.049**
**CRP**	**Peripheral AS**	**1.072(0.928-1.238)**	**0.347**	**0.987(0.813-1.200)**	**0.899**	**1.044(0.881-1.278)**	**0.617**

### 3.2. MR causal analysis

**Fig 2:**
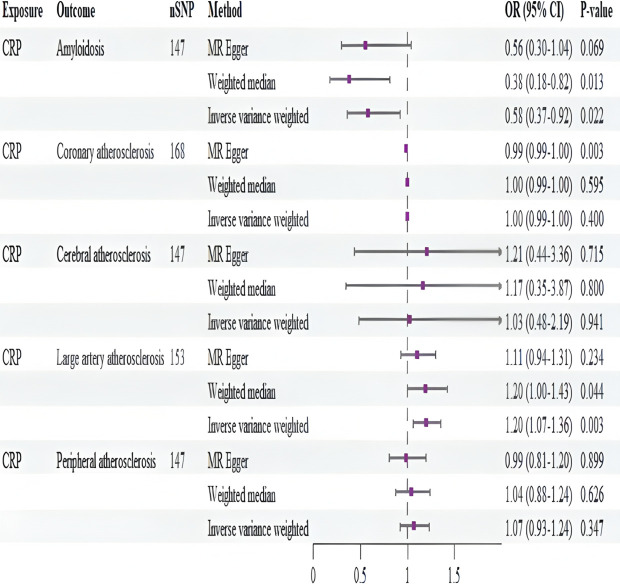
Forest plot of Mendelian randomization results. Forest plot displaying effect estimates (odds ratios, 95% confidence intervals) from Mendelian randomization analyses. Each row represents an outcome (amyloidosis, atherosclerosis subtypes); horizontal lines indicate confidence intervals; diamond symbols represent pooled effect estimates for CRP’s causal effects.

The main method used in this Mendelian causality study was the IVW method, which is a method commonly used to analyze genetic variation to derive reliable causal relationships without the presence of pleiotropy. Positive MR analysis showed a causal relationship between CRP and amyloidosis and between CRP and atherosclerosis of large arteries. The specific results are shown in Table 1. From the data in the table, there was a positive correlation between the risk of developing atherosclerosis of large arteries and CRP levels (IVW:p = 0.003, OR=1.203,95% CI:1.066–1.358). In contrast, a negative correlation was found between the development of amyloidosis and CRP levels, with the risk of amyloidosis decreasing with increasing CRP levels (IVW:p = 0.022, OR=0.582,95% CI:0.366–0.924), generating a correlation forest plot as shown in Fig 2. The present IVW analysis revealed that CRP increased the risk of atherosclerosis by 20.3% (OR=1.203). Notably, the present study found that CRP reduced the risk of amyloidosis, and the correlation was a 41.8% reduction in the risk of amyloidosis (OR=0.582).

### 3.3. MR sensitivity analysis

**Fig 3:**
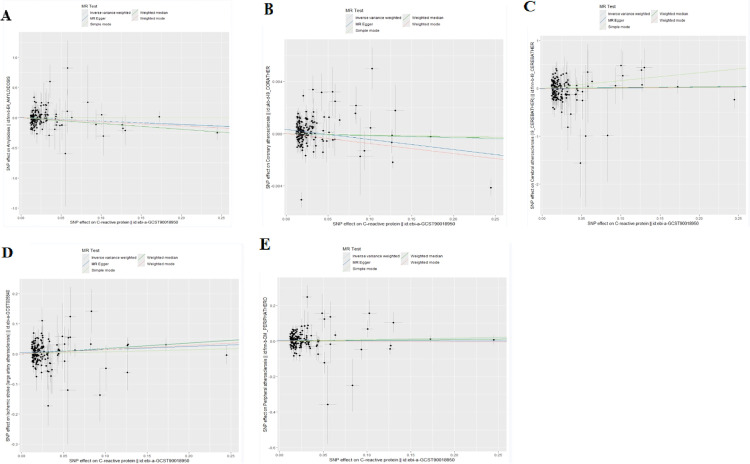
Scatter plots from this Mendelian randomization study. (A) Effect of CRP on amyloidosis; (B) Effect of CRP on coronary atherosclerosis; (C) Effect of CRP on cerebral atherosclerosis; (D) Effect of CRP on aortic atherosclerosis; (E) Effect of CRP on peripheral atherosclerosis. Scatter plots show the causal relationship between CRP (x-axis: genetic predisposition to CRP levels) and outcome risk (y-axis). The regression line slope represents the magnitude of causal effects.

**Fig 4:**
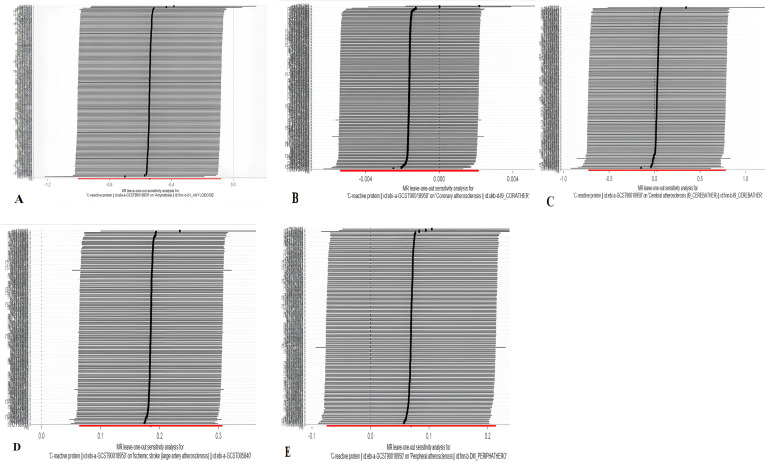
Leave-one-out sensitivity analysis results. (A) Effect of CRP on amyloidosis; (B) Effect of CRP on coronary atherosclerosis; (C) Effect of CRP on cerebral atherosclerosis; (D) Effect of CRP on aortic atherosclerosis; (E) Effect of CRP on peripheral atherosclerosis. Each point indicates the effect estimate after excluding one genetic variant; consistent results across exclusions confirm the robustness of causal associations.

To avoid the influence on the results due to latent level pleiotropy, we also performed sensitivity analysis, mainly MR-Egger, and WM methods, to make the results more reliable and consistent. The heterogeneity of SNPs was assessed by Cochran’s Q test, and no heterogeneity existed if the P value was > 0.05. No evidence of pleiotropy and heterogeneity between exposure and outcome data was found by the analyses of MR-Egger, WM, and Cochran’s Q methods. The associated scatterplot is shown in Fig 3, and the results obtained by the leave-one-out method are shown in Fig 4.

### 3.4. MR reverse analysis

To verify the existence of reverse causality between the two samples, we performed reverse Mendelian randomization analyses with amyloidosis, the four AS as exposures, and CRP as the outcome, respectively, and the results of the analyses did not support evidence of reverse causality, i.e., there were only positive causal associations with each other.

## 4. Discussion

Both amyloidosis and the atherosclerotic (AS) process are associated with inflammation and immune response. Apolipoprotein A-I (ApoA-I) is one of the common factors involved in the pathogenesis of both. Specific gene mutations, such as alterations that affect the amino acid sequence of ApoA-I, may disrupt its normal function and subsequently induce amyloidosis through complex pathophysiological processes [39,40]. Existing studies have demonstrated that amyloidosis differs from AS in terms of the extent of impact and the degree of damage to the cardiovascular system. AS mainly affects the arterial system, whereas amyloidosis can affect blood vessels, tissues, and organs throughout the body. In the cardiovascular aspect, amyloidosis can not only lead to amyloidosis of the epicardial arteries but also cause myocardial cell damage, abnormal cardiac conduction system, and capillary destruction, among other problems [41–45]. The combined effects of these factors result in more severe myocardial ischemia [46]. Although there are certain similarities in their pathogenesis, they have distinct characteristics in disease progression and clinical manifestations. In this Mendelian study, considering comprehensively their significant impacts on the cardiovascular system and the whole body, as well as the commonalities in the pathogenesis related to inflammation and immunity, they are selected as combined outcome variables, aiming to more comprehensively explore the relationships between relevant genetic factors and the occurrence and development of diseases. However, this selection still requires further verification of its rationality through rigorous study design and data analysis.

CRP is closely related to the inflammatory response, and its quantity can increase dramatically during acute infections, and infections with certain bacteria can even lead to a thousand-fold or more increase in its concentration [47]. The protein is a member of the pentameric family of proteins and originates from the same family as serum amyloid A. Both can be used as indicators of inflammation to detect the extent of infection in the body [48,49]. Researchers have found significant individual variability in CRP levels, confirming that this individual variation is related to genetic factors [50,51]. This suggests that CRP can genetically influence individuals, resulting in different responses to inflammatory stimuli. In the inflammatory response, CRP production is mainly stimulated by inflammatory factors, notably IL-6, in addition to IL-1 and tumor necrosis factor-alpha (TNF-α) [52]. As one of the pentameric proteins, it dissociates into monomeric C-reactive protein (mCRP), and its process intermediate mCRPm can exhibit proinflammatory activity by enhancing platelet adhesion and activating the classical complement pathway [53]. The AS process is mediated by inflammatory factors, of which IL-6 and TNF-α are the major ones [54]. In experiments with humans and animals, it was found that CRP is frequently localized to the plaque-forming endothelial complement in humans [55], which is deposited at the lesion site in the endothelium, and C-reactive protein was detected during the course of the disease in rabbits with AS, suggesting that there is a close association between CRP levels and AS [56]. However, there has been controversy among scholars about the role played by CRP in AS. Some studies have shown that CRP binds to LOX-1, a receptor for oxidized low-density lipoprotein (ox-LDL) [57,58], and accumulates mainly in necrotic core areas with high cholesterol content in AS lesions [59]. It has also been found that CRP can be located in foam cells, which are closely associated with the formation of AS [60,61], and binds to enzyme-modified LDL (E-LDL) to impede foam cell formation [62], and it has been hypothesized that CRP may play an inhibitory role in AS formation [63–65]. Relevant meta-analysis data showed that elevated CRP levels can increase the risk of developing cardiovascular disease, leading to worsening cardiovascular events and affecting the prognosis of patients [66,67]. Therefore, this Mendelian randomization study chose to consider AS as an outcome event to clarify the specific causal association between the two.

The present Mendelian randomization study found that there was a causal association between CRP and atherosclerosis, and the relationship between the two was positive, i.e., CRP could increase the risk of atherosclerosis in large arteries. There was no causal relationship with atherosclerosis of the coronary and cerebral arteries, which are common in the cardiovascular system. There is also no causal relationship with atherosclerosis of peripheral arteries. Notably, the Mendelian study conducted by Kuppa et al. in 2023 suggested that there was no causal relationship between CRP and AS. However, our study differed from theirs in terms of sample size. The GWAS data in Kuppa et al.‘s study was sourced from the FinnGen database and was a single sample without specifying the arterial vessels from which the data were obtained [68]. In contrast, in our study, we separately analyzed the four main types of AS according to the occurrence sites of AS. Such differences in the research design might have led to discrepant results, ultimately resulting in different conclusions. The large arteries are responsible for the major blood supply throughout the body, with the aorta playing an important role in transporting blood from the heart to the periphery. An investigation found that in patients who developed atherosclerosis, CRP could better reflect the extent of aortic atherosclerosis compared to coronary arteries, and multivariate analyses showed that the severity of aortic atherosclerosis was an independent factor associated with the level of CRP [69]. This suggests a closer relationship between aortic atherosclerosis lesions and CRP. For the cerebral vasculature, atherosclerosis of large arteries in the skull predisposes to ischaemic stroke. A relevant meta-analysis showed that CRP levels were significantly increased in the acute phase of ischaemic stroke, increasing the risk of dementia in the later phase of stroke [70]. Relevant prospective studies have shown that CRP can act as a predictor of lower limb peripheral atherosclerosis and can be influenced by the upregulation of the inflammatory factor IL-6, which is strongly associated with the progression of AS [71,72]. However, our present study revealed that Mendelian randomization between CRP and atherosclerotic lesions in peripheral arteries was negative, and there was no causal association between the two.

A previous study has shown an association between CRP and amyloidosis. The formation of amyloidosis lesions is associated with amyloid peptides, and by constructing CRP-associated mutants, researchers have found that the mutants almost always bind to amyloid-β peptide and prevent fibril formation, suggesting that CRP can exert an anti-amyloidogenic effect and thus inhibit the amyloidosis process [73]. This clearly indicates that C - reactive protein (CRP) is able to interact with amyloid – associated peptides and intervene in the fibril formation process. In view of the fact that amyloidosis lesions are intricately related to the aggregation and fibril formation of amyloid peptides, the anti – amyloidogenic property of CRP, as revealed by these results, suggests that CRP can inhibit the development of amyloidosis. Notably, our study found a negative effect of CRP on amyloidosis, i.e., CRP reduces the risk of developing amyloidosis, which is in line with speculations made in the previous study. We assessed the association of C-reactive protein on amyloidosis by Mendelian randomization analysis, which can provide better support and ideas for subsequent research and treatment of this disease.

This study, using European-sourced samples, has its limitations in generalizability but offers a unique chance to explore the CRP-amyloidosis relationship in this specific population. The European population’s distinct genetic and environmental traits allow for in-depth investigation of related disease mechanisms. The publicly-sourced aggregated data, though not individual-level, help identify macroscopic associative trends, guiding future research. Methodologically, the innovative use of Mendelian randomization analysis overcomes issues in traditional studies, enabling us to establish a causal link between low CRP levels and amyloidosis, which, despite differing from previous findings, provides new research perspectives. This enriches our understanding of amyloidosis pathogenesis and offers theoretical support for new treatment strategies. Overall, our research, with its unique sample-data handling and methodology, is valuable for advancing knowledge in this area and guiding future research and clinical practice.

When exploring the potential mechanisms between CRP and AS, this study attempted to perform Mendelian randomization (MR) analysis with low-density lipoprotein cholesterol (LDL-C) as a mediator variable, but this was not feasible due to current research limitations. Specifically, identifying genetic instrumental variables (IVs) that meet MR assumptions faced significant challenges:

On one hand, screening for SNPs strongly associated with LDL-C (satisfying an F-statistic > 10) and free of pleiotropy (i.e., influencing AS only through LDL-C without direct effects on other pathways) proved difficult. Most LDL-C-related genetic variants in existing GWAS data (such as rs12916, rs641738, etc.) carry pleiotropic risks, potentially affecting CRP or AS outcomes directly through inflammatory pathways or other mechanisms, leading to biased estimation of mediation effects. On the other hand, linkage disequilibrium (LD) among genetic variants (e.g., r² > 0.001 with distances < 10,000 kb) confounded the independence of IVs, further weakening the analytical power.

Consequently, this study was unable to clarify the mediating role of LDL-C between CRP and AS. Larger-scale and more refined genetic data (such as single-cell sequencing to map causal variants in LD regions) or novel statistical methods (such as multivariate Mendelian randomization) are needed to advance this research.

There are some limitations in this study. The samples were selected from the European population only, which may limit our results in different ethnicities, and the data source is publicly available aggregated statistics rather than originating from individuals. Whether the results obtained are generalizable in the population still needs further research to clarify. Constrained by the existing data, it is particularly noteworthy that the number of cases for cerebral atherosclerosis and amyloidosis is extremely limited. In the context of cerebral atherosclerosis, the dataset encompassed only 104 cases. Small samples are inherently more susceptible to random errors and are prone to producing both false-negative and false-positive outcomes. Likewise, the relatively limited number of amyloidosis cases (n = 226) presents a formidable obstacle. Each case within the dataset has distinct idiosyncrasies. Amyloidosis encompasses diverse disease subtypes, each characterized by unique pathogenic mechanisms, disparate affected organs, and a broad spectrum of symptoms. The pre-amyloidosis health profiles of patients, including comorbid conditions, age, gender, and lifestyle factors, also display substantial heterogeneity. Treatment modalities span a wide gamut, and even within the same disease subtype, genetic backgrounds diverge significantly. Complications during the disease trajectory are variable, and the rate of disease progression differs markedly from one case to another. With a sample of this magnitude, the results are highly vulnerable to these individual differences, potentially leading to substantial deviations between the estimated effect size and the true population parameter. Our results show a causal relationship between the selected exposure factors and outcome events, however, the exact mechanism of influence needs to be confirmed by subsequent experiments.

## 5. Conclusion

In summary, our results validate the negative association between CRP and amyloidosis and the positive association between CRP and AS, which may provide valuable information for the clinical treatment and prevention of the corresponding diseases.

## Supporting information

S1 Appendix STROBE-MR checklist of recommended items to address in reports of Mendelian randomization studies^1.2^. (DOCX)

S1 Table This table presents the genetic data related to the C - reactive protein exposure factor in a Mendelian randomization study. Each row represents information ona genetic variant locus. chr.exposure and pos.exposure indicate the chromosomenumber and locus position respectively; beta.exposure, se.exposure and pval.exposure are the effect estimate, standard error and P - value associated with the exposure factor; samplesize.exposure is the sample size; id.exposure and SNP are identifiers of the genetic variant; effect_allele.exposure and other_allele.exposure are the effect allele and other allele; eaf.exposure is the allele frequency; exposure specifies that the exposure factor is C - reactive protein along with the relevant study identifier; mr_keep.exposure is a boolean variable, presumably indicating whether it is retained for Mendelian randomization analysis; pval_origin.exposure and data_source.exposure explain the source of the P - value and the data source respectively.
(XLSX)
