# Supplementary material for: Mendelian randomization analysis: The causal relationship between C-reactive protein and amyloidosis and between C-reactive protein and atherosclerosis
Source: PLoS One. 2025 Aug 8;20(8):e0329612. doi: 10.1371/journal.pone.0329612 (PMC12334044; doi:10.1371/journal.pone.0329612)
Supplement: S1 Appendix — (DOCX) [file pone.0329612.s001.docx]

**STROBE-MR checklist of recommended items to address in reports of Mendelian randomization studies**^1^ ^2^

| **Item No.** | **Section** | **Checklist item** | **Page No.** | **Relevant text from manuscript** |
| --- | --- | --- | --- | --- |
| 1 | **TITLE and ABSTRACT** | Indicate Mendelian randomization (MR) as the study’s design in the title and/or the abstract if that is a main purpose of the study | 1-2 | Mendelian randomization analysis: the causal relationship between C-reactive protein and amyloidosis and atherosclerosis |
|  | **INTRODUCTION** |  |  |  |
| 2 | **Background** | Explain the scientific background and rationale for the reported study. What is the exposure? Is a potential causal relationship between exposure and outcome plausible? Justify why MR is a helpful method to address the study question | 1 | A number of studies have shown that elevated C-reactive protein levels are linked to the development of atherosclerosis and amyloidosis. However, the exact mechanism by which this connection might be explained is not known. |
| 3 | **Objectives** | State specific objectives clearly, including pre-specified causal hypotheses (if any). State that MR is a method that, under specific assumptions, intends to estimate causal effects | 3-4 | Combined with the above information, CRP can affect the course of amyloidosis and AS lesions. Therefore, we conducted this Mendelian randomization study to explore the specific causal association between CRP and amyloidosis and AS. |
|  | **METHODS** |  |  |  |
| 4 | **Study design and data sources** | Present key elements of the study design early in the article. Consider including a table listing sources of data for all phases of the study. For each data source contributing to the analysis, describe the following: |  |  |
|  | a) | Setting: Describe the study design and the underlying population, if possible. Describe the setting, locations, and relevant dates, including periods of recruitment, exposure, follow-up, and data collection, when available. | 4 | The study methodology complied with the requirements in the STROBE-MR checklist, and three basic assumptions have been fulfilled, i.e., the assumption of correlation, the assumption of exclusivity, and the assumption of independence. |
|  | b) | Participants: Give the eligibility criteria, and the sources and methods of selection of participants. Report the sample size, and whether any power or sample size calculations were carried out prior to the main analysis | 5-6 | The single nucleotide polymorphisms (SNPs) associated with CRP in this study were obtained from the Genome-Wide Association Study (GWAS) meta-analysis dataset provided by the United Kingdom Biobank (UKBB), which can be accessed at https://gwas.mrcieu.ac.uk. A total sample size of 353,466 samples was included in the analysis, which involved 19,057,467 SNPs. Genetic data related to amyloidosis and AS were obtained from the GWAS database, with amyloidosis data from 197,485 individuals (covering 226 cases and 197,259 controls) and 16,380,377 SNPs, and AS Data: coronary atherosclerosis data from 361,194 samples (14,334 cases and 346,860 controls) and 13,860 SNPs. data: coronary atherosclerosis data with 361,194 samples (14,334 cases, 346,860 controls) and 13,586,589 SNPs, cerebral atherosclerosis data with 218,792 samples (104 cases, 218,688 controls) and 16,380,466 SNPs, and aortic atherosclerosis data with 150,765 samples (4,373 cases, 406,111 controls) and 7,992,739 SNPs. The peripheral atherosclerosis data consisted of 168,832 samples (6,631 cases, 162,201 controls) and 16,380,247 SNPs, and the sample sizes in the present study were from European populations |
|  | c) | Describe measurement, quality control and selection of genetic variants | 1 | Mendelian randomization (MR) analysis study. Methods used included inverse variance weighting (IVW), weighted median (WM), MR-Egger method, Cochran's Q, MR-PRESSO, MR-Egger intercept test and leave-one-out sensitivity analysis. |
|  | d) | For each exposure, outcome, and other relevant variables, describe methods of assessment and diagnostic criteria for diseases | 5-6 | Comply with the relevant diagnostic standards for coronary atherosclerosis, cerebral atherosclerosis, aortic atherosclerosis and peripheral atherosclerosis in atherosclerosis |
|  | e) | Provide details of ethics committee approval and participant informed consent, if relevant | 6 | Details of ethics committee approval and participant informed consent are available online from publicly available databases |
| 5 | **Assumptions** | Explicitly state the three core IV assumptions for the main analysis (relevance, independence and exclusion restriction) as well assumptions for any additional or sensitivity analysis | 6-7 | The final desired SNPs were obtained by making P < 5 × 10-8, thus screening the IVs that showed strong association with CRPs after the following process: (1) relevant SNPs were extracted online from the GWAS summary data using the TwoSampleMR package in the R 4.3.2 software, and (2) the genomic distances of the SNP manifestation r2 within 1,000 base pairs (Kb) were greater than 0.001 to address linkage disequilibrium; (3) SNPs with overlapping or alleles that could not be corresponded to were excluded to prevent impact on the analysis results; (4) http://www.phenoscanner.medschl.cam.ac.uk/ was utilized to eliminate the effect of confounding factors [16]; (5) using the statistic F to detect the presence of bias in instrumental variables [17], taking a threshold of F > 10 for identification, with the relevant formula F = [(n-k- 1)/k] * [R2/(1-R2)], where n denotes the sample size and k denotes the IV number. r2 denotes the proportion of genetically related exposure variants. If the F value was less than 10, it indicated that the SNP in question was a weak instrumental variable and was excluded from this analysis to attenuate the bias caused by the results of this study. |
| 6 | **Statistical methods: main analysis** | Describe statistical methods and statistics used |  |  |
|  | a) | Describe how quantitative variables were handled in the analyses (i.e., scale, units, model) | 7-8 | The two-sample two-way Mendelian randomization analysis was used in this study, and the research process was carried out using the TwoSampleMR and MR-PRESSO packages in the R 4.3.2 software, and the main methods used included the inverse variance weighted (IVW) method, the weighted median (WM) method, and the MR-Egger. of which the IVW method is considered to be the causality determination main basis, in which the weighted mean is weighted by the inverse of the variance of each IV to ensure the validity of the IV, and the corresponding P-value <0.05 is considered significant for causality. |
|  | b) | Describe how genetic variants were handled in the analyses and, if applicable, how their weights were selected | 7 | The final desired SNPs were obtained by making P < 5 × 10-8, using the statistic F to detect the presence of bias in instrumental variables, taking a threshold of F > 10 for identification, with the relevant formula F = [(n-k- 1)/k] * [R2/(1-R2)], where n denotes the sample size and k denotes the IV number. |
|  | c) | Describe the MR estimator (e.g. two-stage least squares, Wald ratio) and related statistics. Detail the included covariates and, in case of two-sample MR, whether the same covariate set was used for adjustment in the two samples | 8 | Heterogeneity was assessed by Cochran's Q test, where P < 0.05 indicated the presence of heterogeneity, and a random effects model was applied, and vice versa for a fixed effects model. Causality was assessed using the ratio of ratios (OR) and 95% confidence intervals (CI). If the OR was <1, the exposure was protective of the outcome, and vice versa, it posed a risk to the outcome. At the end of the analysis, we performed a reverse MR analysis with the same parameter settings and data samples. |
|  | d) | Explain how missing data were addressed | 8 | For missing data, consult other databases to supplement them and discard them if necessary. |
|  | e) | If applicable, indicate how multiple testing was addressed | 9 | To avoid the influence on the results due to latent level pleiotropy, we also performed sensitivity analysis, mainly MR-Egger, and WM methods, to make the results more reliable and consistent. The heterogeneity of SNPs was assessed by Cochran's Q test, and no heterogeneity existed if the P value was >0.05. |
| 7 | **Assessment of assumptions** | Describe any methods or prior knowledge used to assess the assumptions or justify their validity | 6-7 | (1) relevant SNPs were extracted online from the GWAS summary data using the TwoSampleMR package in the R 4.3.2 software, and (2) the genomic distances of the SNP manifestation r2 within 1,000 base pairs (Kb) were greater than 0.001 to address linkage disequilibrium; (3) SNPs with overlapping or alleles that could not be corresponded to were excluded to prevent impact on the analysis results; (4) http://www.phenoscanner.medschl.cam.ac.uk/ was utilized to eliminate the effect of confounding factors [16]; (5) using the statistic F to detect the presence of bias in instrumental variables [17], taking a threshold of F > 10 for identification, with the relevant formula F = [(n-k- 1)/k] * [R2/(1-R2)], where n denotes the sample size and k denotes the IV number. |
| 8 | **Sensitivity analyses and additional analyses** | Describe any sensitivity analyses or additional analyses performed (e.g. comparison of effect estimates from different approaches, independent replication, bias analytic techniques, validation of instruments, simulations) | 8 | Heterogeneity was assessed by Cochran's Q test, where P < 0.05 indicated the presence of heterogeneity, and a random effects model was applied, and vice versa for a fixed effects model. Causality was assessed using the ratio of ratios (OR) and 95% confidence intervals (CI). If the OR was <1, the exposure was protective of the outcome, and vice versa, it posed a risk to the outcome. At the end of the analysis, we performed a reverse MR analysis with the same parameter settings and data samples. Finally, we used the Rstudio software platform to visualize the results of the analysis in the form of forest plots, scatter plots, and leave-one-out plots. |
| 9 | **Software and pre-registration** |  |  |  |
|  | a) | Name statistical software and package(s), including version and settings used | 7 | TwoSampleMR package in the R 4.3.2 software |
|  | b) | State whether the study protocol and details were pre-registered (as well as when and where) | 1 | The study procedure was performed with concerning the STROBE-MR checklist. |
|  | **RESULTS** |  |  |  |
| 10 | **Descriptive data** |  |  |  |
|  | a) | Report the numbers of individuals at each stage of included studies and reasons for exclusion. Consider use of a flow diagram | 5 | 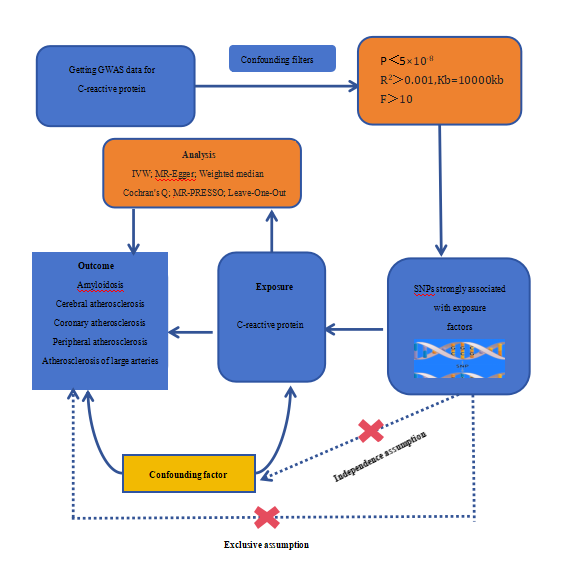 |
|  | b) | Report summary statistics for phenotypic exposure(s), outcome(s), and other relevant variables (e.g. means, SDs, proportions) | 8 | The sample datasets in this study were all from the GWAS database, and the required SNPs were screened as IVs by a P-value less than a threshold of 5 × 10-8, and the F-statistic of each IV was greater than 10, indicating that there was no weak bias in the IV results. |
|  | c) | If the data sources include meta-analyses of previous studies, provide the assessments of heterogeneity across these studies | 9 | No evidence of pleiotropy and heterogeneity between exposure and outcome data was found by the analyses of MR-Egger, WM, and Cochran's Q methods. |
|  | d) | For two-sample MR:  i.  Provide justification of the similarity of the genetic variant-exposure associations between the exposure and outcome samples  ii.  Provide information on the number of individuals who overlap between the exposure and outcome studies | 9 | The main method used in this Mendelian causality study was the IVW method, which is a method commonly used to analyze genetic variation to derive reliable causal relationships without the presence of pleiotropy. Positive MR analysis showed a causal relationship between CRP and amyloidosis and atherosclerosis of large arteries. From the data in the table, there was a positive correlation between the risk of developing atherosclerosis of large arteries and CRP levels (IVW:p=0.003, OR=1.203,95% CI:1.066-1.358). |
| 11 | **Main results** |  |  |  |
|  | a) | Report the associations between genetic variant and exposure, and between genetic variant and outcome, preferably on an interpretable scale | 8 | The sample datasets in this study were all from the GWAS database, and the required SNPs were screened as IVs by a P-value less than a threshold of 5 × 10-8, |
|  | b) | Report MR estimates of the relationship between exposure and outcome, and the measures of uncertainty from the MR analysis, on an interpretable scale, such as odds ratio or relative risk per SD difference | 9 | The main method used in this Mendelian causality study was the IVW method, which is a method commonly used to analyze genetic variation to derive reliable causal relationships without the presence of pleiotropy. Positive MR analysis showed a causal relationship between CRP and amyloidosis and atherosclerosis of large arteries. From the data in the table, there was a positive correlation between the risk of developing atherosclerosis of large arteries and CRP levels (IVW:p=0.003, OR=1.203,95% CI:1.066-1.358). |
|  | c) | If relevant, consider translating estimates of relative risk into absolute risk for a meaningful time period | 9 | Notably, the present study found that CRP reduced the risk of amyloidosis and the correlation was a 41.8% reduction in the risk of amyloidosis (OR=0.582). |
|  | d) | Consider plots to visualize results (e.g. forest plot, scatterplot of associations between genetic variants and outcome versus between genetic variants and exposure) | 11-13 | For specific display results, please see the relevant figures in the text. |
| 12 | **Assessment of assumptions** |  |  |  |
|  | a) | Report the assessment of the validity of the assumptions | 9 | No evidence of pleiotropy and heterogeneity between exposure and outcome data was found by the analyses of MR-Egger, WM, and Cochran's Q methods. |
|  | b) | Report any additional statistics (e.g., assessments of heterogeneity across genetic variants, such as *I^2^*, Q statistic or E-value) | 9 | To avoid the influence on the results due to latent level pleiotropy, we also performed sensitivity analysis, mainly MR-Egger, and WM methods, to make the results more reliable and consistent. The heterogeneity of SNPs was assessed by Cochran's Q test, and no heterogeneity existed if the P value was >0.05. |
| 13 | **Sensitivity analyses and additional analyses** |  |  |  |
|  | a) | Report any sensitivity analyses to assess the robustness of the main results to violations of the assumptions | 9 | No evidence of pleiotropy and heterogeneity between exposure and outcome data was found by the analyses of MR-Egger, WM, and Cochran's Q methods. |
|  | b) | Report results from other sensitivity analyses or additional analyses |  | None |
|  | c) | Report any assessment of direction of causal relationship (e.g., bidirectional MR) | 14 | To verify the existence of reverse causality between the two samples, we performed reverse Mendelian randomization analyses with amyloidosis, the four AS as exposures, and CRP as the outcome, respectively, and the results of the analyses did not support evidence of reverse causality, i.e., there were only positive causal associations with each other. |
|  | d) | When relevant, report and compare with estimates from non-MR analyses |  | See the text for specific results. |
|  | e) | Consider additional plots to visualize results (e.g., leave-one-out analyses) | 13 | For specific display results, please see the relevant figures in the text. |
|  | **DISCUSSION** |  |  |  |
| 14 | **Key results** | Summarize key results with reference to study objectives | 16-17 | The present Mendelian randomization study found that there was a causal association between CRP and atherosclerosis, and the relationship between the two was positive, i.e., CRP could increase the risk of atherosclerosis in large arteries. There was no causal relationship with atherosclerosis of the coronary and cerebral arteries, which are common in the cardiovascular system. There is also no causal relationship with atherosclerosis of peripheral arteries. The large arteries are responsible for the major blood supply throughout the body, with the aorta playing an important role in transporting blood from the heart to the periphery. An investigation found that in patients who developed atherosclerosis, CRP could better reflect the extent of aortic atherosclerosis compared to coronary arteries, and multivariate analyses showed that the severity of aortic atherosclerosis was an independent factor associated with the level of CRP [44]. This suggests a closer relationship between aortic atherosclerosis lesions and CRP. For the cerebral vasculature, atherosclerosis of large arteries in the skull predisposes to ischaemic stroke. A relevant meta-analysis showed that CRP levels were significantly increased in the acute phase of ischaemic stroke, increasing the risk of dementia in the later phase of stroke [45]. Relevant prospective studies have shown that CRP can act as a predictor of lower limb peripheral atherosclerosis and can be influenced by the upregulation of the inflammatory factor IL-6, which is strongly associated with the progression of AS [46, 47]. However, our present study revealed that Mendelian randomization between CRP and atherosclerotic lesions in peripheral arteries was negative and there was no causal association between the two. |
| 15 | **Limitations** | Discuss limitations of the study, taking into account the validity of the IV assumptions, other sources of potential bias, and imprecision. Discuss both direction and magnitude of any potential bias and any efforts to address them | 17-18 | There are some limitations in this study, the samples were selected from the European population only, which may limit our results in different ethnicities, and the data source is publicly available aggregated statistics rather than originating from individuals, whether the results obtained are generalizable in the population still needs further research to clarify. Our results show a causal relationship between the selected exposure factors and outcome events, however, the exact mechanism of influence needs to be confirmed by subsequent experiments. |
| 16 | **Interpretation** |  |  |  |
|  | a) | Meaning: Give a cautious overall interpretation of results in the context of their limitations and in comparison with other studies | 16-17 | The present Mendelian randomization study found that there was a causal association between CRP and atherosclerosis, and the relationship between the two was positive, i.e., CRP could increase the risk of atherosclerosis in large arteries. There was no causal relationship with atherosclerosis of the coronary and cerebral arteries, which are common in the cardiovascular system. There is also no causal relationship with atherosclerosis of peripheral arteries. The large arteries are responsible for the major blood supply throughout the body, with the aorta playing an important role in transporting blood from the heart to the periphery. An investigation found that in patients who developed atherosclerosis, CRP could better reflect the extent of aortic atherosclerosis compared to coronary arteries, and multivariate analyses showed that the severity of aortic atherosclerosis was an independent factor associated with the level of CRP [44]. This suggests a closer relationship between aortic atherosclerosis lesions and CRP. For the cerebral vasculature, atherosclerosis of large arteries in the skull predisposes to ischaemic stroke. A relevant meta-analysis showed that CRP levels were significantly increased in the acute phase of ischaemic stroke, increasing the risk of dementia in the later phase of stroke [45]. Relevant prospective studies have shown that CRP can act as a predictor of lower limb peripheral atherosclerosis and can be influenced by the upregulation of the inflammatory factor IL-6, which is strongly associated with the progression of AS [46, 47]. However, our present study revealed that Mendelian randomization between CRP and atherosclerotic lesions in peripheral arteries was negative and there was no causal association between the two. |
|  | b) | Mechanism: Discuss underlying biological mechanisms that could drive a potential causal relationship between the investigated exposure and the outcome, and whether the gene-environment equivalence assumption is reasonable. Use causal language carefully, clarifying that IV estimates may provide causal effects only under certain assumptions | 14 | Both amyloidosis and the AS process are associated with inflammation and immune response. ApoA-I is a common factor involved in the pathogenesis of both and can be genetically mutated by substituting sequences in amino acids, which results in the deletion of the normal gene locus to induce amyloidosis]. This suggests that the two are related to each other. Amyloidosis is more serious than AS, which mainly affects the arteries, whereas amyloidosis can affect blood vessels, tissues, and organs all over the body. For the cardiovascular system, amyloidosis of the heart leads to more severe myocardial ischemia, which is caused by amyloidosis of epicardial arteries and the destruction of capillarie. Due to the similarities in their pathogenesis and effects on tissues and organs, this Mendelian study chose to use them together as an outcome variable. |
|  | c) | Clinical relevance: Discuss whether the results have clinical or public policy relevance, and to what extent they inform effect sizes of possible interventions |  |  |
| 17 | **Generalizability** | Discuss the generalizability of the study results (a) to other populations, (b) across other exposure periods/timings, and (c) across other levels of exposure | 17 | There are some limitations in this study, the samples were selected from the European population only, which may limit our results in different ethnicities, and the data source is publicly available aggregated statistics rather than originating from individuals, whether the results obtained are generalizable in the population still needs further research to clarify. Our results show a causal relationship between the selected exposure factors and outcome events, however, the exact mechanism of influence needs to be confirmed by subsequent experiments. |
|  | **OTHER INFORMATION** |  |  |  |
| 18 | **Funding** | Describe sources of funding and the role of funders in the present study and, if applicable, sources of funding for the databases and original study or studies on which the present study is based | 18 | This study was supported by the Natural Science Foundation of Jilin Province (Project No. YDZJ202401067ZYTS) project. |
| 19 | **Data and data sharing** | Provide the data used to perform all analyses or report where and how the data can be accessed, and reference these sources in the article. Provide the statistical code needed to reproduce the results in the article, or report whether the code is publicly accessible and if so, where | 18 | The data and information related to this study have been recorded in the tables and supplementary materials of this article, please consult the authors if you have any questions. |
| 20 | **Conflicts of Interest** | All authors should declare all potential conflicts of interest | 18 | The authors declare that there are no commercial or financial conflicts of interest in the results of this research. |

This checklist is copyrighted by the Equator Network under the Creative Commons Attribution 3.0 Unported (CC BY 3.0) license.

1. Skrivankova VW, Richmond RC, Woolf BAR, Yarmolinsky J, Davies NM, Swanson SA, et al. Strengthening the Reporting of Observational Studies in Epidemiology using Mendelian Randomization (STROBE-MR) Statement. JAMA. 2021;under review.

2. Skrivankova VW, Richmond RC, Woolf BAR, Davies NM, Swanson SA, VanderWeele TJ, et al. Strengthening the Reporting of Observational Studies in Epidemiology using Mendelian Randomisation (STROBE-MR): Explanation and Elaboration. BMJ. 2021;375:n2233.
